# Expansion of the exotic macroalga *Batophora occidentalis* in *Posidonia oceanica* meadows and other native benthic habitats

**DOI:** 10.1371/journal.pone.0338173

**Published:** 2026-07-20

**Authors:** Andrea Anton, Silvia Paoletti, Lidia Cucala-Garcia, Carlos Morell, Sara Muñiz-Quintana

**Affiliations:** Global Change Research Group, IMEDEA (CSIC-UIB), Mediterranean Institute for Advanced Studies, Esporles, Illes Balears, Spain; University of Ferrara: Universita degli Studi di Ferrara, ITALY

## Abstract

Marine invasive macroalgae can be detrimental to native ecosystems. The exotic green macroalga *Batophora occidentalis* was first reported in 2020 in Estany des Peix lagoon in Formentera (Spain). In 2023 and 2024, our surveys revealed that it had spread to 100% of the surveyed locations within the lagoon. In 2024, *B. occidentalis,* reached an average cover of 30% in *Cymodocea nodosa* meadows and 26% in *Caulerpa prolifera* meadows, with respective biomasses of 52 g m ⁻ ^2^ and 44 g m ⁻ ^2^, making it the most abundant macrophyte in these two native benthic habitats. We also found exotic *B. occidentalis* growing epiphytically on the leaves and rhizomes of *Posidonia oceanica* within the lagoon, with an average cover of 12% in 2023 and 16% in 2024, and a biomass of 3 g m ⁻ ^2^ in 2024. Notably, it covered nearly 25% of the *P. oceanica* leaf surface and reached up to five times the weight of individual leaves. Alarmingly, we report the presence of a *B. occidentalis* specimen on a *P. oceanica* shoot in a nearby meadow outside the lagoon, signalling a potential spread beyond its current range. Additionally, the exotic macroalgae colonized various natural and hard substrates, including boat hulls, as it was growing on nearly 20% of boats anchored in the only marina in the lagoon. With this study, we aim to encourage prompt action from local and regional governments. We may be witnessing the early stages of a broader expansion of this exotic alga, highlighting a critical moment to implement early management measures such as monitoring, and, where possible, containment within the lagoon.

## Introduction

The introduction of species is an increasingly significant environmental challenge, with an estimated 35% rise in the number of emerging exotic species globally by 2050 [1,2]. The Mediterranean Sea, with more than 1,000 reported exotic species [3], is the most invaded marine region in the world [4]. In this region, marine exotics have significant ecological impacts on biodiversity [5] and on native species [6–9], including endemic taxa [6,9,10]. This high rate of introductions is driven by various factors, including the opening of the Suez Canal in 1869, increased shipping activity, and accidental introductions from aquaculture facilities [4]. Among these introductions, marine exotic macrophytes are responsible for some of the largest environmental impacts on native species [11], primarily through negative effects on native primary producers [12]. For example, the invasive macroalgae *Lophocladia lallemandii* can increase the mortality of seagrass *Posidonia oceanica* (Linnaeus) Delile, 1813 shoots by 2.5 to 5 times [4]. The Mediterranean Sea is home to an estimated 118 introduced macrophyte species, including macroalgae and seagrasses, with an average of 22 new introductions per decade since 1990 [13]. One such recent introduction is the green macroalga *Batophora occidentalis* (Harvey), 1998 (Chlorophyta: Dasycladales).

This exotic macroalgae was first detected in the Mediterranean in the spring of 2020 in the Estany des Peix (Formentera, Balearic Islands, Spain) [14], and its distribution was subsequently updated in 2023. The genus *Batophora* is native to the tropical and subtropical western Atlantic [14]. Outside its native range, it was first reported in the Canary Islands (Spain) between 1990–1992 [15] and later in the Chesapeake Bay (USA) in 2015 [16]. In the Mediterranean, an additional record of the genus comes from the Mar Menor (Murcia, southern Spain) [17]. The two Mediterranean detections of *B. occidentalis* occurred almost simultaneously: in Formentera in May 2020 and Mar Menor in November 2021, with specimens from both introductions having overlapping morphological traits [14,17]. It has been hypothesized that both populations may originate from the same introduction event [17], with shipping, particularly luxury yachts that overwinter in the Caribbean and travel to the Mediterranean in the summer, proposed as the most likely pathway of introduction to Formentera [14].

The genus *Batophora* comprises three recognized taxa: *Batophora oerstedii, Batophora occidentalis* and *Batophora occidentalis* var. *largoensis* [18]. These taxa are taxonomically distinguishable based on morphological characteristics, including algal size, the location of the whorls along the main axis, the location and size of the gametophores, and the size and shape of the gametangia [18]. However, there is no definitive scientific consensus on how these traits define the species, which complicates species identification. Terradas-Fernández et al. (2022) identified the *Batophora* species in Mar Menor as either *Batophora occidentalis* or *Batophora occidentalis* var. *largoensis* based on morphology. Similarly, Hall and Schneider (2023) reported the introduction of *Batophora oerstedii* in the Chesapeake Bay using morphological traits. At the Estany des Peix (Formentera), the exotic macroalgae was tentatively identified as *Batophora occidentalis* var. *largoensis* based on morphological traits; however, the identification was considered inconclusive [14]. Therefore, in this study we refer to the specimens found in Formentera as *B. occidentalis*.

Here, we describe the extent and ecological characteristics of the *B. occidentalis* invasion in Estany des Peix, Formentera, 3–4 years after its initial report [14]. Specifically, we: 1) assessed the presence of the exotic macroalgae around the perimeter (and nearby locations outside) of the lagoon during the late summers of 2023 and 2024, 2) documented the presence of *B. occidentalis* with photographs on various habitats, including natural substrates, artificial structures, and boats, 3) quantified by recording with video cameras the *B. occidentalis* benthic cover in two locations (a *P. oceanica* meadow and sandy bottom) inside the lagoon in 2023, and in four locations in 2024 (a meadow of *Cymodocea nodosa* (Ucria) Ascherson, 1870, a meadow of *Caulerpa prolifera* (Forsskål) J.V. Lamoroux, 1809, and two *P. oceanica* meadows located inside and outside the lagoon, respectively), 4) measured the biomass of *B. occidentalis* growing on the blades of these three macrophyte species and/or in the surrounding sediment within the meadows in 2024, and 5) quantified the presence of the exotic macroalgae attached to the hulls of the boats anchored in the main marina of the lagoon.

## Methods

**Fig 1:**
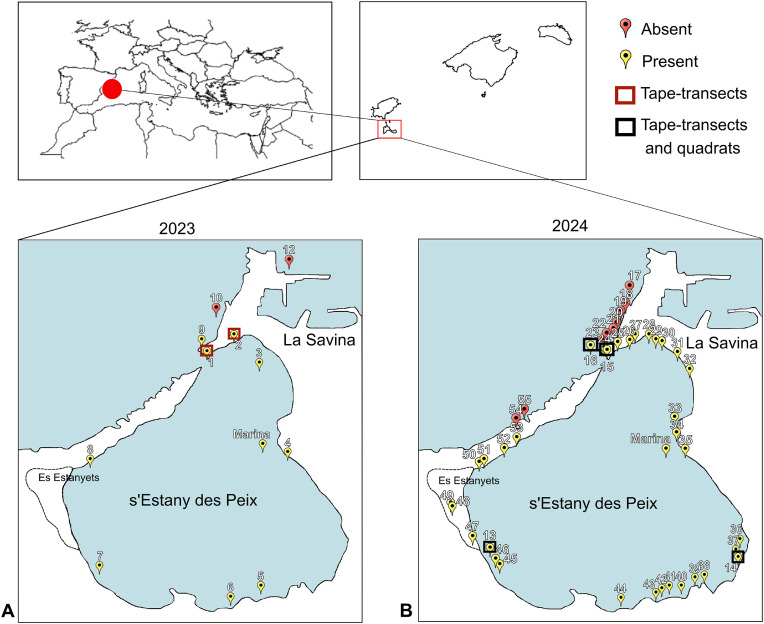
Map of the study site, the Estany des Peix lagoon, in Formentera Island (Balearic Islands, Spain) in A) 2023 and B) 2024. Points (1-55) indicate the presence (green) or absence (red) of *Batophora occidentalis* assessed visually in eight locations inside the lagoon and four locations outside the lagoon in 2023, and 34 locations inside the lagoon and 10 locations outside the lagoon in 2024, where pictures were also taken. In 2023, locations 1 and 2 (red squares) were recorded with tape-transects for % benthic cover. In 2024, locations 13-16 (blue squares) were recorded with tape-transects for % benthic cover and sampled with quadrats for biomass analysis. Map outlines were manually digitized from geographical boundaries of Natural Earth data (www.naturalearthdata.com), redrawn and simplified by the authors. The coordinates of the sample sites were measured in the field using GPS.

To assess the distribution of *B. occidentalis* around the perimeter of Estany des Peix lagoon (38.72618, 1.41200; Formentera Island, Spain; Fig 1), visual surveys were conducted on September 27^th^ and 28^th^ 2023 and 2^nd^ – 3^rd^ of October 2024. A total of 12 and 44 study locations were surveyed in 2023 and 2024, respectively. In 2023, eight locations were surveyed inside the lagoon (locations 1–8; Fig 1A), one at the lagoon entrance (location 9; Fig 1A), and three outside the lagoon (locations 10–12; Fig 1A). Within the lagoon, seven sites were located around the perimeter at depths of < 1 meter, while one site (location 3; Fig 1A) was surveyed at approximately 2 meters depth. In 2024, 34 locations were surveyed around the perimeter of the lagoon and 10 outside the lagoon (locations 13–55; Fig 1B), all at depths < 1.5 meters. At each location, approximately a 50-meter stretch was visually inspected to determine the presence of *B. occidentalis*. Environmental parameters were measured at a subset of sites. In 2023, temperature was recorded at two locations (locations 1, and 2; Fig 1A. In 2024, temperature, salinity, and dissolved oxygen (mg l^-1^ and %) were measured in three study locations (locations 13–15; Fig 1B) using a ProSolo (YSI Xylem) handheld probe. In 2024, we quantified the number of boats moored in the Estany des Peix marina with the exotic algae growing on their hulls by visually inspecting all vessels from the dock.

We quantified the percent benthic cover of *B. occidentalis* and the main habitat-forming macrophytes using the point intersect method along transect lines [19–21]. In 2023, we surveyed two study locations (location 1 and 2, Fig 1A) corresponding to a sandy bottom habitat and a *P. oceanica* meadow. We deployed six and two 10-meter-long transect tapes at each site respectively, and conducted video recordings along each transect using a GoPro Hero 7 camera. Similarly in 2024, we quantified benthic cover at four study locations (location [13–16], Fig 1B) corresponding to three benthic habitats inside the lagoon (*P. oceanica*, *C. nodosa* and *C. prolifera* meadows) and one habitat outside the lagoon (a *P. oceanica* meadow). At each location, three 10 m transects were deployed and recorded. For each transect, the timestamp was noted, and a video of 98 ± 17 seconds was taken while hovering over the transect at a close distance. In the laboratory, benthic cover was quantified at every 10 cm mark along each transect (n = 100 points per transect). The seafloor was classified in one the following categories: (1) meadow (either *C. prolifera*, *C. nodosa* or *P. oceanica*, depending on the habitat), (2) *B. occidentalis* on sediments, (3) *B. occidentalis* growing on leaves or fronds of living macrophytes, (4) dead matte, (5) rock or (6) sand. Based on observations from quadrat samples (see below), the invasive alga was classified as occurring on sediments when it was independently attached to the substrate (category 2), even if in close proximity to other species, or as occurring on blades when attached to macrophyte aboveground structures (category 3). When classified as growing on leaves or fronds (category 3), the point was recorded only once, despite also implying coverage by the underlying meadow, to ensure that total cover summed to 100%. Dead matte represented the substrate formed by the residual rhizomes, roots, and subsurface parts of *P. oceanica* following the loss of the aboveground canopy. Both sides of the 10 cm mark line were considered to aid classification; however, when underlying cover differed between sides, the left side was used. When the underlying cover changed due to frame perspective or when image quality was reduced by movement or glare, annotations were made only when the mark line was in focus and/or positioned at the centre of the frame. Coverage percentages were calculated as follows:


$$\begin{array}{c} Species\;coverage\;(\%)=\;\frac{n\;Species}{\text{1}\text{0}\text{0}}*\text{1}\text{0}\text{0} \end{array}$$

Total coverage of *B. occidentalis* was calculated as the sum of its coverage on sediments and on leaves. Total meadow coverage was calculated as the sum of meadow and *B. occidentalis* on leaves or fronds, as the latter indicates the presence of underlying meadow.

In 2024, macrophyte density and biomass per unit area were assessed using quadrat-based methods [22,23]. Macrophytes within three 10 x 10 cm quadrat were collected at the 1, 5, and 9 m points on each transect of the four study locations (totalling 36 quadrat samples). Samples were collected into zip-lock bags and conserved at −18°C until processing. For each quadrat sample, meadow density was estimated from shoot counts, except for the *C. prolifera* habitat, where total number of fronds was recorded. Specimens were sorted, and biomass was determined as wet weight and dry weight biomass following standard methods [21,22]. Wet weight was measured after pat drying samples on absorbent paper using a precision balance (resolution 100 µg) and dry weight was determined after oven drying at 60°C for several days until samples appeared completely dry [6,24,25]. Biomass was assessed separately for 1) unimpacted meadow biomass, 2) impacted meadow biomass (i.e., with *B. occidentalis* on leaves), 3) *B. occidentalis* biomass attached independently to sediments, and 4) *B. occidentalis* biomass on blades. The total number of *B. occidentalis* stalks and the proportion of fertile ones were noted for both above categories (3 and 4). In *C. nodosa* and *P. oceanica* habitats, the number of leaves per shoot was also noted [22]. For the *P. oceanica* habitat, shoots were processed individually. For each invaded leaf, we recoded the position within the shoot (chronologically from the centre, following [23]), total length, and minimum attachment height of *B. occidentalis*. The way rhizoids attached on the macrophyte tissue (directly on blades, or on epiphytes) was noted. Representative specimens were photographed using an Olympus Tough TG-5 camera, and examined under a stereomicroscope and a ZEISS Axio Zoom V16 digital microscope.

All statistical analysis were done using R. Statistical analyses were mainly performed on data from 2024 as in 2023 data only included coverage of two habitats, one of which (sand habitat) was not surveyed in 2024. Meadow and *B. occidentalis* coverages in *P. oceanica* habitat in 2023 and 2024 were compared using a t-test. The differences in mean coverage among biogenic habitats were compared in 2024 with a one-way ANOVA test followed by a post-hoc Tukey HSD test. When ANOVA assumptions (Kozak and Piepho, 2017) were not met, non-parametric Kruskal-Wallis test followed by Dunn’s test were used instead. The differences in biomass among benthic habitats in 2024 were compared using a linear mixed model (*lme()* function using package *nlme*) with transect ID as random effect, followed by a post-hoc Tukey HSD (*emmeans()* function using package *emmeans*) [26]. Residual plots were used to check for violations of normality on residuals (Schielzeth et al., 2020). The relationships between continuous variables were explored using linear regression modelling (*lm()* function using package *stats*). Surveys were conducted under authorization issued by the Direcció General de Medi Natural i Gestió Forestal, Conselleria d’Agricultura, Pesca i Medi Natural, Govern de les Illes Balears (A04013554).

## Results

**Fig 2:**
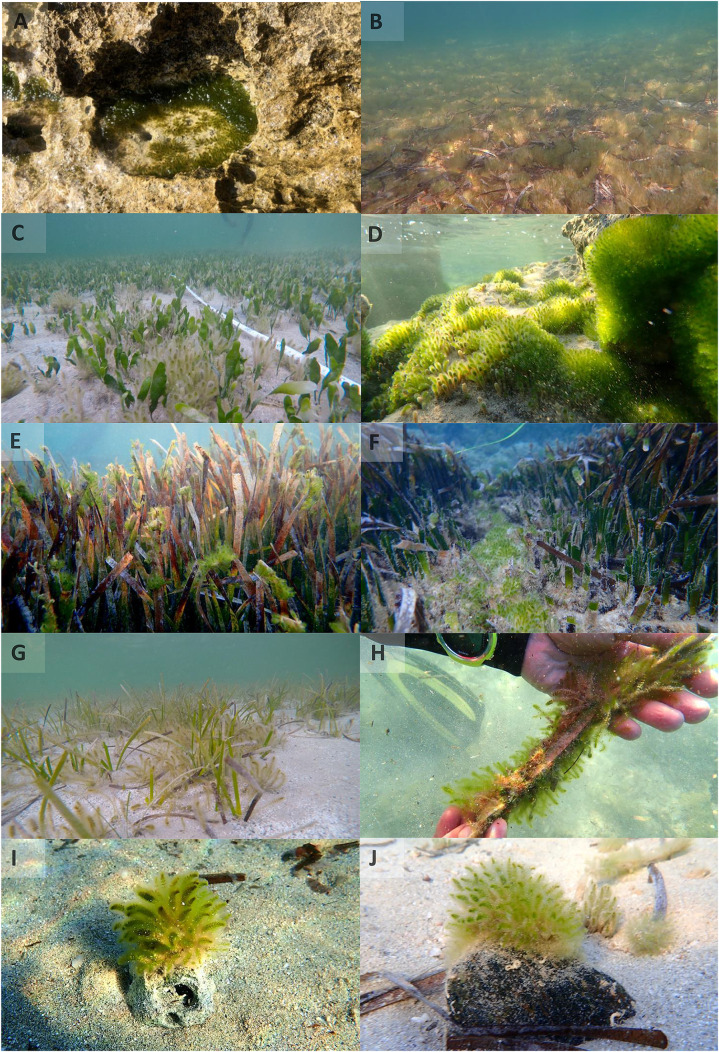
Exotic macroalgae *Batophora occidentalis* (A) growing on rock pools outside the lagoon, (B) forming extensive meadows growing mainly on *Posidonia oceanica* detrital leaves (2 m depth approx.). (C) growing on meadows of the macroalgae *Caulerpa prolifera*, (D) proliferating on pebbles and rocky outcrops, (E) growing on the leaves of the seagrass *P. oceanica*, (F) expanding on the rhizome of sparse areas within *P. oceanica* meadows, (G) proliferating on meadows of the seagrass *Cymodocea nodosa*, (H) growing on detrital leaves of *P. oceanica*, and (G-H) growing on natural hard substrates in sandy areas (e.g., old sea shells; G and H) in the Estany des Peix in the island of Formentera in September 2023 and October 2024. Photo credit: Andrea Anton.

The average temperature in the lagoon during the surveys was 26.5 in 2023 and 25.1 ^º^C in 2024. In 2024, mean salinity and dissolved oxygen were 38.9 ppt and 7.6 mg l^-1^ (or 116% saturation), respectively. *B. occidentalis* was present in all surveyed locations along the perimeter of the lagoon in 2023 and 2024 at <1 m depth (Fig 1A and B), as well as at the additional site surveyed within the lagoon in 2023 at approximately 2 m depth (location 3; Fig 1A). The species was also observed at the lagoon entrance in rock pools in 2023 (location 9; Fig 2A), but was not detected there during the 2024 surveys (location 16; Fig 1B). No individuals of *B. occidentalis* were observed at any of the surveyed locations outside the lagoon in either year (locations 10–12 in 2023 and locations 16, 18–23, 54, and 55 in 2024; Fig 1A and B).

**Fig 3:**
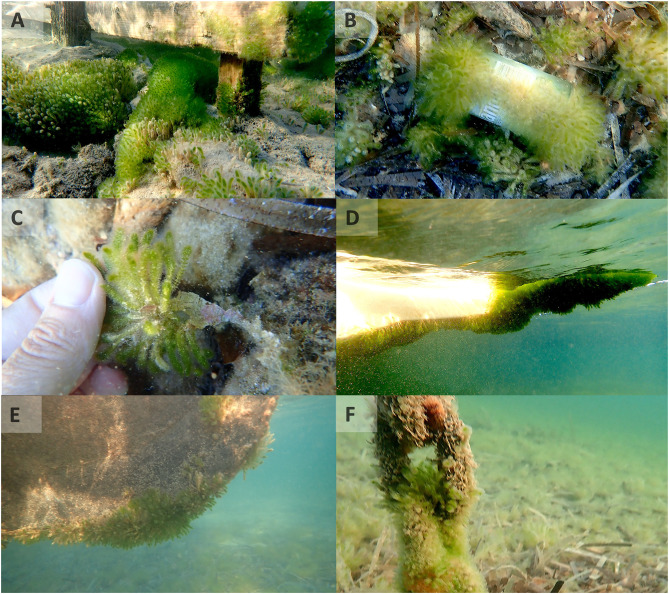
Exotic macroalgae *Batophora occidentalis* growing on man-made structures such as wooden harbours (A), marine debris (e.g., soda can; B), boat anchor chains (C) and boat hulls (D) in the Estany des Peix in the island of Formentera in September 2023. Photo credit: Andrea Anton.

*B. occidentalis* was observed growing across a variety of natural habitats (Fig 2) including the leaves and rhizomes of healthy seagrass *P. oceanica* meadows (Fig 2E and F), detrital leaves of *P. oceanica* (Fig 2H), sediments within *C. nodosa* meadows (location 5; Fig 2G), as well as rocks and rubble (Fig 2D). It was also found attached to other biogenic hard substrates scattered across sandy bottoms, such as seashells (Fig 2I, and J). The species was likewise common on artificial substrates, including wooden harbour structures (Fig 3A), marine debris (e.g., cans, plastic pieces; Fig 3B), boat anchor chains (Fig 3C), and boat hulls (Fig 3D). Notably, *B. occidentalis* was recorded on 17.4% (11 out of 63) of the boat hulls anchored in the main marina of the lagoon (38.727519, 1.415702). Additionally, floating clumps of *B. occidentalis* were observed throughout the lagoon.

Transect-tape surveys conducted in 2023 revealed a widespread distribution of *B. occidentalis* within the lagoon, both sand and a *P. oceanica* meadows, with mean coverages of 15.8 ± 7.3% and 17.5 ± 14.8%, respectively (Fig 5A, S2 Table, Table 1 and [38]). In sandy areas (62.8 ± 15.0% sand coverage; location 1; Fig 1A and Table 1), *B. occidentalis* was mainly attached to detached debris and detrital *P. oceanica* leaves. Within the *P. oceanica* meadow inside the lagoon (location 1; Fig 1A), both the *P. oceanica* coverage and the percentage of leaves containing *B. occidentalis* in 2023 were similar to that of 2024 (t-test; p = 0.1225 and p = 0.3311; respectively), with 12.0 ± 12.7% in 2023 and 16.3 ± 3.2% in 2024 of the latter (Table 1).

**Table 1:** Tape-transects mean ± SD coverage percentage (% summing to 100) in 2023 and 2024 of native seagrass/seaweed species, exotic *Batophora occidentalis* on sediments or blades, and remaining sediment types for each benthic habitat (n = 12).

Habitat type	Year	Location	Native macrophyte	*B. occidentalis* on sediments	*B. occidentalis* on blades	*P. oceanica* matte	Rock	Sand
Sand bottom	2023	Lagoon	0.0 ± 0.0	15.8 ± 7.3	0.0 ± 0.0	21.3 ± 9.2	0.0 ± 0.0	62.8 ± 15.0
*Posidonia oceanica*	2023	Lagoon	29.5 ± 23.3	5.5 ± 2.1	12.0 ± 12.7	3.5 ± 4.9	32.5 ± 19.1	17.0 ± 14.1
*Caulerpa prolifera*	2024	Lagoon	18.0 ± 11.5	25.7 ± 4.7	0.0 ± 0.0	0.0 ± 0.0	2.3 ± 2.1	54.0 ± 8.2
*Cymodocea nodosa*	2024	Lagoon	15.3 ± 6.5	30.0 ± 3.6	0.0 ± 0.0	0.0 ± 0.0	0.0 ± 0.0	54.7 ± 9.3
*Posidonia oceanica*	2024	Lagoon	54.7 ± 3.2	0.7 ± 0.6	16.3 ± 3.2	8.0 ± 1.7	13.7 ± 2.5	6.7 ± 1.2
*Posidonia oceanica*	2024	Open sea	81.7 ± 6.8	0.0 ± 0.0	0.0 ± 0.0	10.6 ± 6.5	1.3 ± 1.5	6.3 ± 3.8

**Fig 4:**
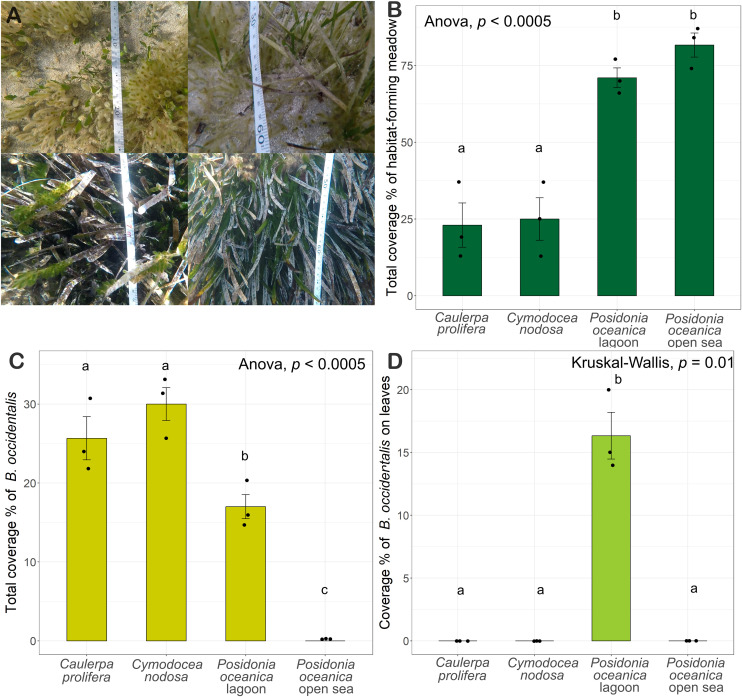
*Batophora occidentalis* and main macrophyte % benthic coverage from tape-transects in 2024. **A)** Examples of each benthic habitat sampled by video recordings; **top left**: *Caulerpa prolifera* habitat (location 13 on Fig 1B), **top right**: *Cymodocea nodosa* habitat (location 14 on Fig 1B), **bottom left**: *Posidonia oceanica* habitat inside the lagoon (location 15 on Fig 1B), and **bottom right**: *P. oceanica* habitat outside the lagoon in the open sea (location 16 on Fig 1B). Barplots of (**B**) total meadow coverage (%), (**C**) total *B.* coverage (%), and (**D**) coverage (%) of *B. occidentalis* on leaves, in each benthic habitat in 2024. The results of the one-way ANOVA and of Kruskal-Wallis test are shown, while letters indicate statistically significant differences in the means tested by post-hoc pairwise comparison using Tukey HSD and Dunn’s test, respectively. Black dots indicate raw data in panels B, C and D.

In 2024, tape-transects indicated that total meadow coverage across benthic habitats ranged from 23% to 82%, with *C. prolifera* covering 23 ± 12.5% of the seafloor, *C. nodosa* 25 ± 12%, *P. oceanica* inside the lagoon 71 ± 5.6%, and *P. oceanica* in the open sea 81.7 ± 6.8% (Fig 4B, Table 1, and [38]). *B. occidentalis* was present in three out of four locations (Fig 4C), being absent from *P. oceanica* meadows outside the lagoon. Its coverage reached 25.7 ± 4.7% in *C. prolifera* habitats, 30.0 ± 3.6% in *C. nodosa habitats*, and 17.0 ± 2.7% in *P. oceanica* habitats inside the lagoon, exceeding the coverage of the native habitat-forming species in the first two cases (Fig 4B and Table 1). Post hoc Tukey HSD tests indicated significant differences in total *B. occidentalis* coverage among habitats, except between *C. prolifera* and *C. nodosa* (Fig 4C). In *P. oceanica* meadows, *B. occidentalis* was observed exclusively on leaves, with coverage significantly lower than the other habitats (*p* < 0.0005, one-way ANOVA; Table 1, Fig 4D).

**Fig 5:**
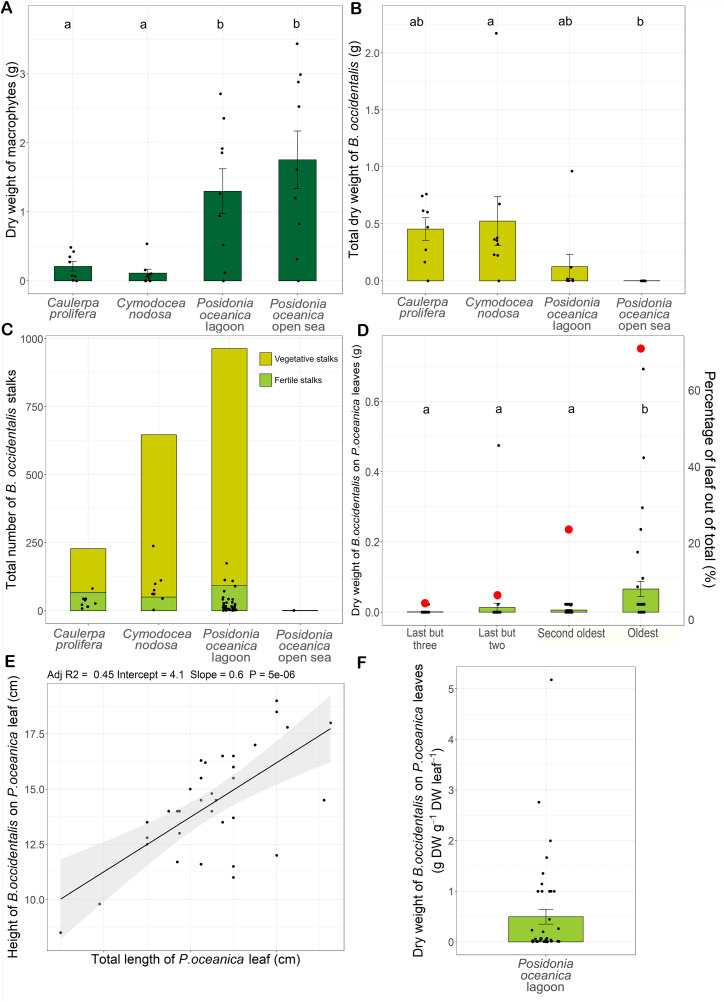
Differences in mean meadow biomass (A), mean total *Batophora occidentalis* (B), and sum of *B. occidentalis* vegetative and fertile (with gametophores) stalks among biogenic habitats in 2024 (C). **D**) Boxplot distribution of *B. occidentalis* biomass of *Posidonia oceanica* leaves inside the lagoon as a function of leaf position in the shoot (left y-axis). The superimposed red points indicate the proportion (%) of leaves with *B. occidentalis* for each position among the total number of shoots covered with *B. occidentalis* (right y-axis). **E**) Relationships between the height of *B. occidentalis* attachment on the leaf and the total length of the latter. **F**) Epiphytic load of *B. occidentalis* on leaves expressed in g DW g^-1^ DW leaf. Barplots show the results of the linear mixed models with letters indicating statistically significant differences in the means, while the scatterplot shows the results of the linear regression model. Black dots indicate raw data in all panels.

In 2024, *B. occidentalis* was found in all quadrat samples collected inside the lagoon (location 12–15; Fig 1B) except for one sample within *C. nodosa* habitat. Outside the lagoon, in the open sea, only a single *B. occidentalis* stalk was found on one *P. oceanica* leaf (location 16; Fig 1B and S3 Fig). Total biomass of *B. occidentalis* irrespective of attachment type, differed between *C. nodosa* and *P. oceanica* habitats in the open sea (*p* = 0.03, Tukey’s test). The biomass of *P. oceanica*, both inside the lagoon and in the open sea, were significantly higher than that of *C. prolifera* and *C. nodosa* inside the lagoon (Tukey’s test, Fig 5A). In *C. prolifera* and *C. nodosa* habitats, *B. occidentalis* was predominantly attached directly to sediments, where it reached higher biomass than the native habitat-forming species (Table 2, Fig 5B). The size of *B. occidentalis* bundles varied across samples (Table 2), with the largest (in both biomass and number of stalks) observed in *C. nodosa* habitat (Fig 5C). However, bundles in *C. prolifera* habitat had a higher proportion of fertile stalks (23.2 ± 15.6%) compared to *C. nodosa* (15.9 ± 21.4%) and *P. oceanica* habitats (2.5 ± 5.3%). In *P. oceanica* habitat within the lagoon, *B. occidentalis* was almost exclusively attached to seagrass leaves. Its biomass on leaves ranged from 0 to 5.2 g g ⁻ ¹ DW, with a mean of 0.50 ± 0.96 g g ⁻ ¹ DW (Fig 5F). Attachment to other macrophyte species was rare and observed only four times: once on *C. nodosa*, twice on *C. prolifera*, and once on a *Halimeda tuna* specimen found while sampling the *C. prolifera* location. Due to their rarity, these cases are documented only as photographic evidence in the Supplementary Material (S4 Fig).

**Table 2:** Summary of data (mean ± SD) obtained from the quadrant samples analysis in 2024. Meadow density is presented as n° shoots/0.01 m^2^, except for *Caulerpa prolifera* habitat which is expressed in n° leaves/0.01 m^2^. Biomass is presented as dry weight in g/0.01 m^2^. *Batophora occidentalis* biomass is presented separately as biomass independently hooked on the sediments (per sample) and biomass on leaves (per leaf), respectively. The sample size available for computing each average value is indicated below.

Habitat type	Location	Meadow density	Meadow biomass	N° leaves per shoot	Shoot biomass	*B. occidentalis* on sediments biomass	*B. occidentalis* on sediments n° stalks (fertile)	*B. occidentalis* on blades biomass	*B. occidentalis* on leaves n° stalks (fertile)	*B. occidentalis* biomass on covered leaves
*Caulerpa prolifera*	Lagoon	17.38 ± 15.29 (n = 8)	0.21 ± 0.20 (n = 8)	–	–	0.44 ± 0.28 (n = 8)	42 ± 23 (10 ± 6) (n = 8)	0.04 ± 0.05 (n = 2)	15 ± 7 (3 ± 4) (n = 2)	0.04 ± 0.05 (n = 2)
*Cymodocea nodosa*	Lagoon	3.56 ± 3.68 (n = 9)	0.11 ± 0.17 (n = 9)	4.04 ± 0.98 (n = 23)	0.12 ± 0.13 (n = 7)	0.52 ± 0.64 (n = 9)	99 ± 65 (7 ± 5) (n = 8)	< 0.0005 (n = 1)	3 (2) (n = 1)	< 0.0005 (n = 1)
*Posidonia oceanica*	Lagoon	10.12 ± 5.77 (n = 9)	1.30 ± 0.98 (n = 9)	6.05 ± 2.13 (n = 78)	0.13 ± 0.09 (n = 78)	0.002 ± 0.007 (n = 9)	49 (3) (n = 1)	0.03 ± 0.08 (n = 44)	25 ± 37 (2 ± 7) (n = 44)	0.06 ± 0.09 (n = 44)
*Posidonia oceanica*	Open sea	11.33 ± 6.52 (n = 9)	1.75 ± 0.25 (n = 9)	6.00 ± 1.85 (n = 102)	0.15 ± 0.13 (n = 102)	0.00 ± 0.00 (n = 9)	–	< 0.0005 (n = 1)	1 (0) (n = 1)	0.02 (n = 1)

In the *P. oceanica* meadow within the lagoon, *B. occidentalis* was primarily attached to the oldest (71% of cases) and second oldest (22% of cases) leaves of the shoots (Fig 5D), with a significantly higher biomass on the oldest leaf (*p* = 0.0002, Tukey’s test) than the subsequent younger leaves. However, in many cases where *B. occidentalis* occurred on the second oldest leaf, the oldest leaf was broken and shortened, suggesting that it may have previously hosted the alga before losing its tip. *B. occidentalis* was consistently found at the apex of the leaf, with attachment height significantly correlated with leaf length (Adj-R^2^ = 0.45, *p* < 0.0005, linear regression model; Fig 5E).

**Fig 6:**
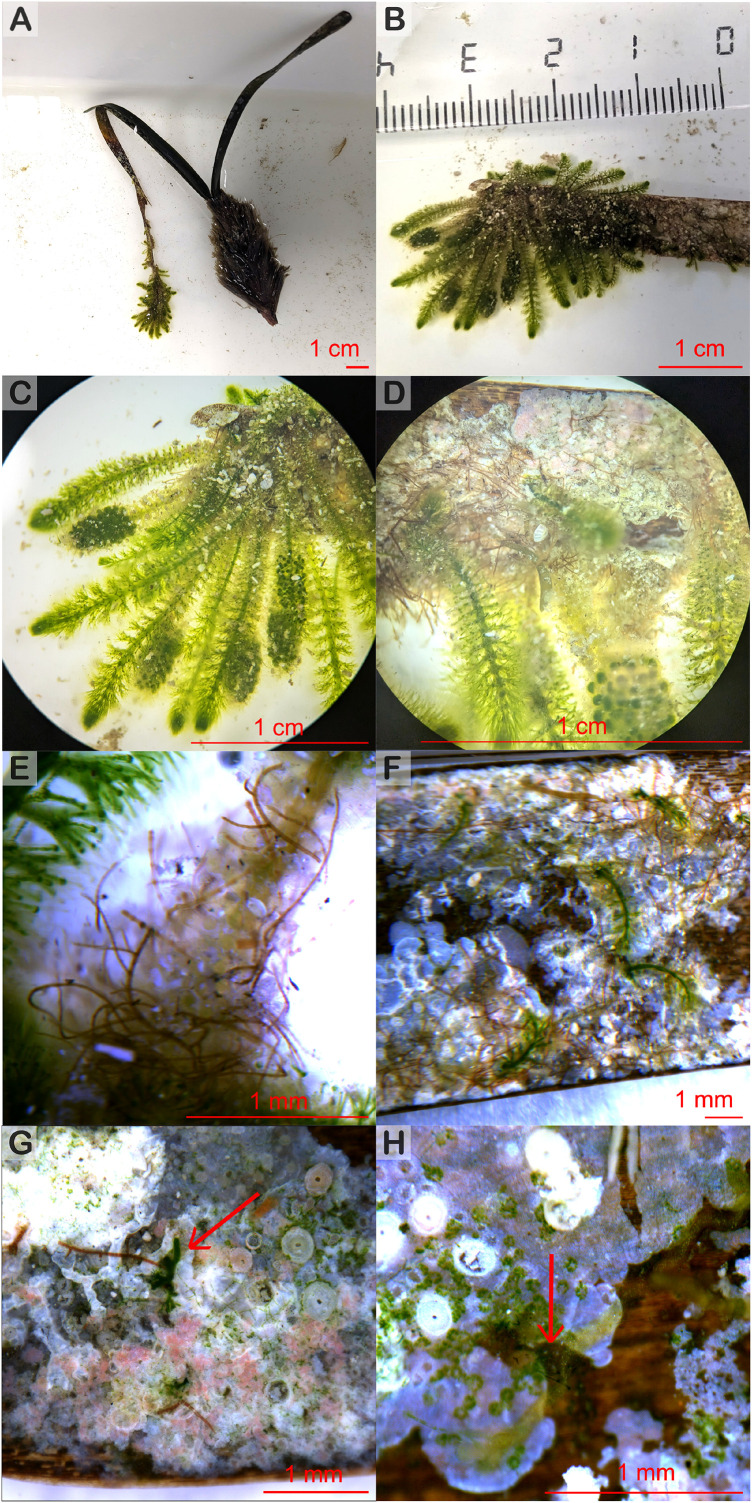
Progressive close-up of *Batophora occidentalis* development and attachment on *Posidonia oceanica* leaves. **A–D**) *B. occidentalis* consists of a bundle of green stalks and a brown rhizoid system that anchors onto the epiphytes present on *P. oceanica* leaves, primarily attaching near the top, where most of its biomass is concentrated. Stalks can be distinguished in vegetative ones (without gametophores) and fertile ones (with spherical dark-green gametophores). **E**) Sedimentation between the stalks occurs due to their fuzzy nature. **F-H**) Lower along the leaf fewer and smaller stalks are present, but an extensive rhizoid system may help the formation of additional stalks. The formation of new rhizoid and stalks – indicated by red arrows – starts from spot-like green patches. Photo credit: Silvia Paoletti.

Closer inspection of colonized *P. oceanica* leaves revealed that the leaf tips were covered by a dense network of *B. occidentalis* brown rhizoids from which the vegetative (free of spherical gametophores) and fertile (with spherical gametophores) stalks emerged (Fig 6). The rhizoid system and the stalk bases were observed to attach primarily to epiphytes growing on the leaf surface. Due to the fluffy nature of the stalks, sediment particles were frequently trapped within the bundles. In the sheath of the leaves, fewer and smaller stalks were observed, although the rhizoid network remained relatively extensive wherever epiphytes were present (Fig 6 A to D). The bottom panels of Fig 6 (E-H) highlighted how an array of spot-like green patches not visible to the naked eye seem to be the precursor of new rhizoids and stalks in the lower parts of the leaf.

## Discussion

We detected a widespread presence of *B. occidentalis* in the Estany des Peix lagoon in both 2023 and 2024, across all studied locations and habitats, including *P. oceanica* seagrass meadows. Notably, we report for the first time its epiphytic growth on the leaves and rhizomes of *P. oceanica* (Fig 2E and 2F), where it reached nearly 20% benthic coverage within this endemic habitat. The epiphytic growth of this exotic macroalgae might have detrimental effects on endemic *P. oceanica*. Most introduced macroalgae in the Mediterranean do not grow epiphytically on the blades of native macrophytes (e.g., seagrasses and macroalgae) but instead spread over sediment or colonize rhizomes in sparse meadows, as observed for *Caulerpa racemosa* var. *cylindracea* on *P. oceanica* meadows [27,28]. An exception is the exotic rhodophyte *Lophocladia lallemandii*, which also grows epiphytically on *P. oceanica* leaves and has been shown to increase seagrass mortality by 2.5–5 times compared to non-invaded sites [6].

In Formentera, the exotic *B. occidentalis* is abundant on *P. oceanica* leaves, covering nearly 25% of the leaf surface at all canopy levels, from the rhizomes to the blade tips of the seagrass (Fig 2E, 2F, and Fig 6). This extensive coverage may substantially reduce irradiance within the canopy. A comparable effect has been reported for *L. lallemandii*, which attenuates light within the *P. oceanica* canopy, thereby limiting seagrass growth and reducing shoot size and internode length [4]. Additionally, the epiphytic growth of *B. occidentalis* could damage *P. oceanica* leaves by increasing the epiphytic load. Seagrass leaves with heavy epiphyte loads are known to become more prone to breakage when loads reach 0.8–1.5 g g ⁻ ^1^ DW, and especially above 1.5 g g ⁻ ^1^ DW [29]. In Estany des Peix, the epiphytic load of *B. occidentalis* on *P. oceanica* leaves ranged from 0 to 5.2 g g ⁻ ^1^ leaf ⁻ ^1^ DW, with 27% of leaves exceeding 0.8 g g ⁻ ^1^ DW and 9% exceeding 1.5 g g ⁻ ^1^ DW. Field observations indicated that many heavily colonized leaves (Fig 4A and Fig 6A) were bent, folded, or partially torn, likely due to excess weight. Moreover, many detached *P. oceanica* leaves were found on the lagoon floor, often partially or fully covered by *B. occidentalis* (Fig 2B and 2H). Whether colonization by *B. occidentalis* occurred before or after leaf detachment remains to be determined.

The dense growth of *B. occidentalis* on rhizomes and matte in sparse *P. oceanica* areas, reaching 16.3% cover in 2024 (Fig 2F), raises concerns for the native seagrass species. For example, the invasive *L. lallemandii* also colonizes rhizomes and leaves at meadow edges and low-density *P. oceanica* patches, leading to reduced seagrass shoot size, lower leaf biomass, and reduced percentage of living shoots [27]. Similarly, sparse seagrass beds and meadow edges are negatively affected by the invasive green macroalga *C. cylindracea* [26]. For both of these invasive species, the ability to attach to and overgrow *P. oceanica* rhizomes and leaves seems crucial in their invasion process [27,30]. Like *C. cylindracea* and *Caulerpa taxifolia*, which use rhizoids to attach to the seagrass tissues [27], *B. occidentalis* has a holdfast with rhizoids that facilitates attachment to structures [17]. This mechanism, potentially enhanced by the presence of epiphytic communities on *P. oceanica* leaves, may enable *B. occidentalis* to establish at the leaf apex and subsequently spread downwards (Fig 6).

Quite alarmingly, we found a stalk of *B. occidentalis* growing on a leaf of *P. oceanica* at location outside the lagoon (S4 Fig). Based on our sampling effort, this corresponds to an estimated density of nearly 12 colonized shoots m^-2^ in the adjacent *P. oceanica* meadow outside the lagoon. In 2023, *B. occidentalis* was also found inside three small rock pools at the lagoon entrance. Although *Batophora* species are eurythermal and euryhaline in its native range and can occupy a variety of habitats [31], their distribution tends to be concentrated in sheltered, low-exposure environments, likely in relation to wave energy [32]. This pattern was consistent with the initial detection of *B. occidentalis* in Formentera [13]. However, over time, the species may expand to other suitable areas beyond the lagoon. Such spread would pose a significant threat to the *P. oceanica* meadows of the Pityusic Islands, which are among the most extensive and well-preserved in the Mediterranean and are designated UNESCO World Heritage Site [33,34]. The potential presence of *B. occidentalis* in other areas around the Pityusic Islands is undetermined, nevertheless fundamental for documenting the ongoing expansion.

Estany of Peix (Formentera) is a sandy-bottom lagoon characterized by extensive accumulations of detrital *P. oceanica* leaves in the northwestern sector, as well as meadows of *C. prolifera* and *C. nodosa* distributed throughout the lagoon and a *P. oceanica* meadow at the entrance on the lagoon (Dantart et al. 1990). In the southern part of the lagoon (location 5 and 14; Fig 1A and 1B), *B. occidentalis* was observed growing on the sediment of *C. nodosa* meadows, where it became the dominant macrophyte in terms of both percentage cover and biomass (S2 Fig, Fig 5A and 5B, Fig 6A and 6B). Previous studies have documented negative ecological effects of exotic macrophytes on *C. nodosa* canopies. For instance, in Sicily, the exotic seagrass *Halophila stipulacea* reduced the shoot density of native *C. nodosa* year-round by forming a dense rhizome mats that outcompete native rhizomes, potentially displacing them down into anoxic sediment layers [35,36]. Similarly, in the Gulf of Naples, invasive *C. cylindracea* has been shown to impair the photosynthetic performance of *C. nodosa* through the phytotoxic effects of the secondary metabolite caulerpenyne produced by the invasive macroalga [37]. Although limited, we also observed *B. occidentalis* growing on *C. nodosa* leaves (Table 1 and S5 Fig). Given that *C. nodosa* leaves are smaller than those of *P. oceanica* (leaf surface of 9 and 83 cm^2^, respectively; [38]), they are likely less able withstand heavy epiphytic loads of *B. occidentalis*, potentially leading to increased leaf breakage or detachment. Additionally, *C. nodosa* has a shorter leaf lifespan (~55 days; [39,40]) than *P. oceanica* (202–345 days; Hemminga and Duarte 2000), which may limit the time available for *B. occidentalis* to establish and grow on its leaves. In contrast, in the Mar Menor lagoon in Murcia, Terradas-Fernández et al. (2022) found no *B. occidentalis* in *C. nodosa* meadows, suggesting that *B. occidentalis* may face challenges colonizing dense seagrass canopies. However, our observations in Estany des Peix differ, as *B. occidentalis* was abundant in *C. nodosa* meadows, reaching mean cover approximately twice that of the native seagrass (30% vs 15.3%, respectively in 2024; Table 1).

Similarly, *B. occidentalis* dominated both in percentage cover and biomass within *C. prolifera* meadows in the eastern part of the lagoon (location 7 and 13; Fig 1A and 1B, S2 Fig, Fig 5A and 5B, Fig 6A and 6B). On two occasions, *B. occidentalis* was found growing on the rhizome and fronds of *C. prolifera*; however, in both cases, the host plants appeared to be in poor health condition (S4 Fig). The limited colonization of *C. prolifera* by *B. occidentalis* might be explained by several factors. *C. prolifera* exhibits rapid growth and high regenerative capacity, allowing it to outcompete native seagrasses for space in lagoonal environments [41,42]. Additionally, *Caulerpa* species produce caulerpenyne, a secondary metabolite that serves as a chemical defence against herbivory [31], further enhancing their competitive advantage for space. The combination of chemical defence, fast growth and a short blade turnover may limit the overgrowth of *C. prolifera* by the exotic species.

To our knowledge, this study provides the first quantitative record of benthic coverage and abundance of *B. occidentalis* worldwide. In all surveyed lagoon habitats other than *P. oceanica* meadows, *B. occidentalis* was the dominant macrophyte in terms of both benthic coverage and biomass. Its cover exceeded that of native species, reaching values approximately twice those of *C. nodosa* (30% *B. occidentalis* vs 15.3% *C. nodosa* in 2024), and nearly twice those of *C. prolifera* (25.7% *B. occidentalis* vs 18% *C. prolifera*, respectively in 2024), while also being the main macrophyte on bare sand habitats 15.8% *B. occidentalis* in 2023). At times, the spread of exotic benthic algae can lead to extensive substrate coverage, with cascading effects on biodiversity, native assemblages, and trophic interactions, often resulting in habitat homogenization and ecosystem degradation [7,43]. For example, *Caulerpa cylindracea* and *Womersleyella setacea* invaded a range of Mediterranean habitats becoming the main macrophyte species causing habitat impoverishment [43]. In an extreme example, the brown algae *Rugulopteryx okamurae* has exceeded 85% coverage in parts of the Strait of Gibraltar [44] and reached 100% coverage in areas of the North-western Mediterranean [7]. The high coverage values of *B. occidentalis* observed at Estany des Peix are therefore concerning, as they suggest potential for this species to dominate benthic substrates over time.

*B. occidentalis* was also observed growing on several natural hard substrates, including rocks, pebbles, and empty shells of gastropods and bivalves (Fig 2). Similarly, [45] reported its presence across all sediment types, from muddy sand to rocks, along Estany of Peix perimeter, while [17] documented its occurrence throughout the north-eastern margin of Mar Menor, attached to substrates such as pebbles and mollusk shells (e.g., *Hexaplex trunculus* and *Pinna nobilis*). In the Chesapeake Bay (USA), introduced *Batophora oerstedii* has also been reported growing on shells in very shallow water (<20 cm depth) [16]. However, it remains unclear whether *B. occidentalis* can colonize living hard-shell species such as gastropods or bivalves. If so, this could have negative implications for these organisms, as epibiont fouling is known to impair bivalve functional traits [46]. In addition to natural substrates, *B. occidentalis* was frequently found on artificial structures in Estany des Peix, including wooden peers (Fig 3), plastic debris, and metallic substrates such as cans, boat anchor chains, and hulls (Fig 3), consistent with observations from other regions [16,17,45]. Notably, *B. occidentalis* was recorded on growing on almost 20% of the hulls of the boats in the main marina of the lagoon in 2024. This is of particular concern, as small recreational vessels may act as vectors to spread *B. occidentalis* to other marinas and coastal areas in the Mediterranean. Hull fouling is a primary pathway for the global transport of invasive species [47]. Indeed, [48] found that 71% of recreational vessels boats in the Mediterranean hosted at least one exotic species (up to 11 per vessel) and that many of these boats travelled to distant marinas where such species were not yet present, highlighting their role as vectors for biological invasions.

Some species of exotic macroalgae are easily identified by their morphological attributes. For instance, exotic *Codium fragile* can be distinguished from the native *Codium decorticatum* by the presence of apiculate utricle tips visible in cross sections [49]. In the case of *B. occidentalis*, the species can be distinguished from the native relative *Dasycladus vermicularis* by features such as the spacing between whorls and the distribution of gametophores in the whorl branchlets [17]. However, these morphological criteria are insufficient to reliably distinguish among species within the genus *Batophora*. Populations of *B. occidentalis* in Formentera and Mar Menor have been suggested to belong to the same species, and potentially originate from the same introduction event, due to overlapping morphological traits [17]. Specimens from Formentera were tentatively identified as *Batophora occidentalis* var*. largoensis* based on morphological descriptions [14]. However, [16] were unable to confirm species-level or varietal identity due to the limited availability of molecular data for this genus. Further genetic investigation is therefore required to accurately resolve the taxonomy of these populations, which is essential for understanding their ecology and invasion dynamics.

Primary producers are ranked one of the most damaging group of marine exotics based on their quantified ecological impacts, often exerting negative effects on other primary producers [11,12,50]. In this study, we document for the first time the exotic *B. occidentalis* growing on the leaves and rhizomes of the endangered seagrass *P. oceanica* meadows, as well as within meadows of *C. nodosa* and *C. prolifera*. The most severe impacts of exotic species occur when they replace ecosystem engineers [51]. The endemic seagrass *P. oceanica* is a key engineering species in the Mediterranean Sea [52], covering an estimated area of 1.2 million hectares [53]. Its meadows provide essential ecosystem services, including habitat provision for marine biodiversity, sediments stabilization, and carbon sequestration, and are recognized as priority habitats under the European Habitats Directive (92/43/CEE). Similarly, meadows of *C. nodosa* provide relevant ecosystem functions, acting as nursery and feeding grounds, reducing coastal erosion and supporting nutrient cycling and carbon sequestration [54]. Finally, *C. prolifera* also play a key role, particularly in lagoon systems, by facilitating the sinking and removal of dissolved inorganic nitrogen and enhancing resistance to eutrophication [55]. In the present study, we provide evidence of potential ecological threats posed by *B. occidentalis* to these important native biogenic habitats.

Four years after its first detection in the Estany des Peix, *B. occidentalis* is now widely distributed across all benthic habitats within the lagoon, with observations suggesting a potential spread beyond its boundaries. While eradication is often proposed for localized invasions [56], successfull stories in marine ecosystems remain rare (e.g., *Spartina alterniflora*; [57]). Although *B. occidentalis* in Formentera is currently confined to this small lagoon (approximately 2 km²), it has achieved extensive coverage, with an average density of 5,400 stalks per square meter. Under these conditions, complete eradication may not be feasible. However, targeted management actions could still be effective. Removal efforts focused on the lagoon entrance may help limit natural spread, while measures addressing human-mediated dispersal are essential. Given the high occurrence of *B. occidentalis* on boat hulls, periodic and mandatory cleaning of biofouling in vessels operating within Estany des Peix should be considered to reduce the risk of secondary spread. Key ecological information necessary for effective management, such as its reproductive cycle and seasonal growth seasonal patterns, remains unknown and requires further investigation. Nonetheless, we propose two urgent actions. First, we call for intensified research and monitoring efforts on *B. occidentalis*, particularly in lagoonal systems and marinas across the Balearic Islands for early detection. Second, we urge local and regional governments implement immediate management action by prioritizing the removal of *B. occidentalis* from boat hulls within the lagoon and targeted removal near the lagoon inlet to prevent further colonization in Formentera and other Mediterranean regions.

## Supporting information

S1 Fig Coverage percentage (%) in 2023 for each category for each transect assessed in sand and *Posidonia oceanica* habitats. (PDF)

S2 Fig Coverage percentage (%) in 2024 for each category for each transect and benthic habitat assessed. (PDF)

S3 Fig A *Batophora occidentalis* stalk growing on a leaf of *Posidonia oceanica* from the quadrant samples collected from the meadows outside the lagoon in the open sea. Photo credit: Silvia Paoletti.
(PDF)

S4 Fig Four cases where *Batophora occidentalis* was found attached on leaves of macrophyte species other than *Posidonia oceanica.* A) Specimen found anchored to the stolon of *Caulerpa prolifera* (indicated with a red arrow); B) specimen found attached to a leaf of *Caulerpa prolifera*; C) specimen found attached to a leaf of *Halimeda tuna* within the *C. prolifera* meadows; and D) specimen found attached to a leaf of *Cymodocea nodosa*. Photo credit: Silvia Paoletti.
(PDF)
