# Supplementary material for: Expansion of the exotic macroalga Batophora occidentalis in Posidonia oceanica meadows and other native benthic habitats
Source: PLoS One. 2026 Jul 20;21(7):e0338173. doi: 10.1371/journal.pone.0338173 (PMC13384322; doi:10.1371/journal.pone.0338173)

FIGURE S1. Coverage percentage (%) in 2023 for each category for each transect assessed in sand and *Posidonia oceanica* habitats.

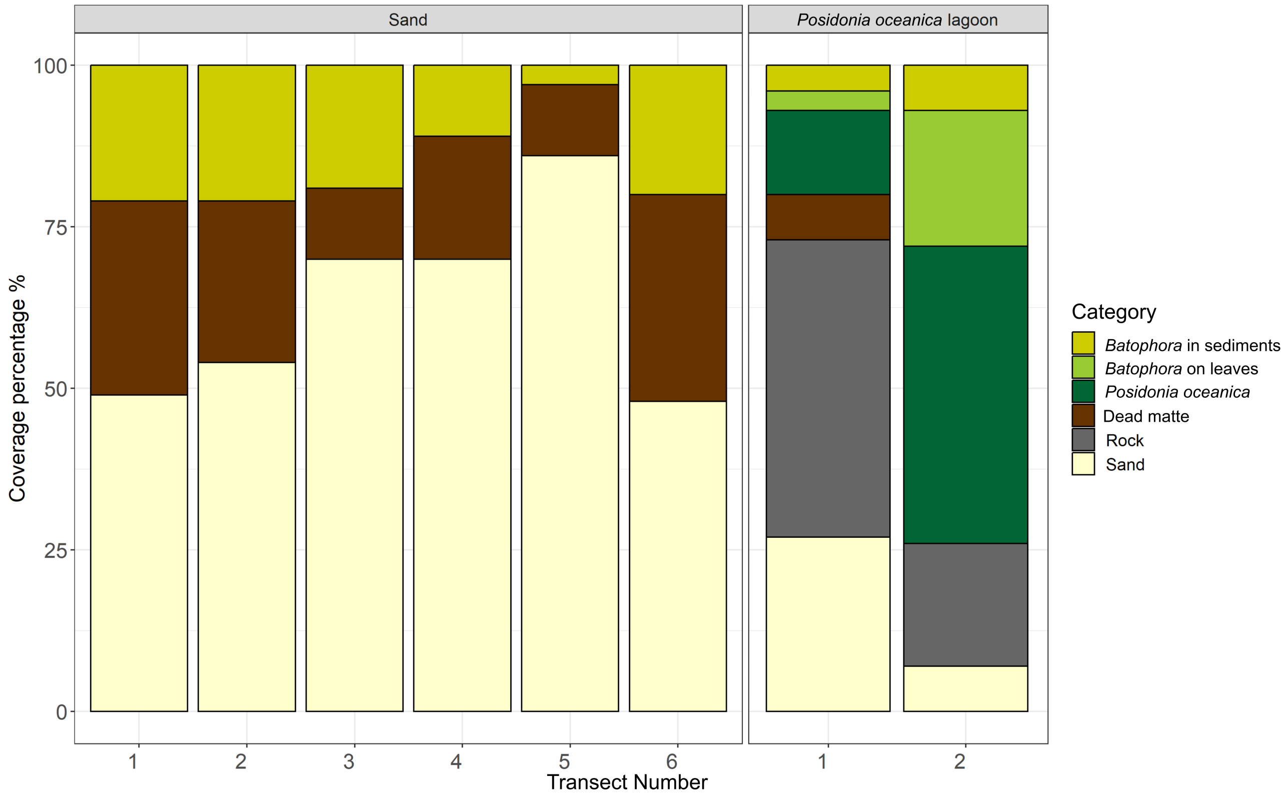

Supplement: S1 Fig — (PDF) [file pone.0338173.s001.pdf]
