# Supplementary material for: Expansion of the exotic macroalga Batophora occidentalis in Posidonia oceanica meadows and other native benthic habitats
Source: PLoS One. 2026 Jul 20;21(7):e0338173. doi: 10.1371/journal.pone.0338173 (PMC13384322; doi:10.1371/journal.pone.0338173)

FIGURE S2. Coverage percentage (%) in 2024 for each category for each transect and benthic habitat assessed.

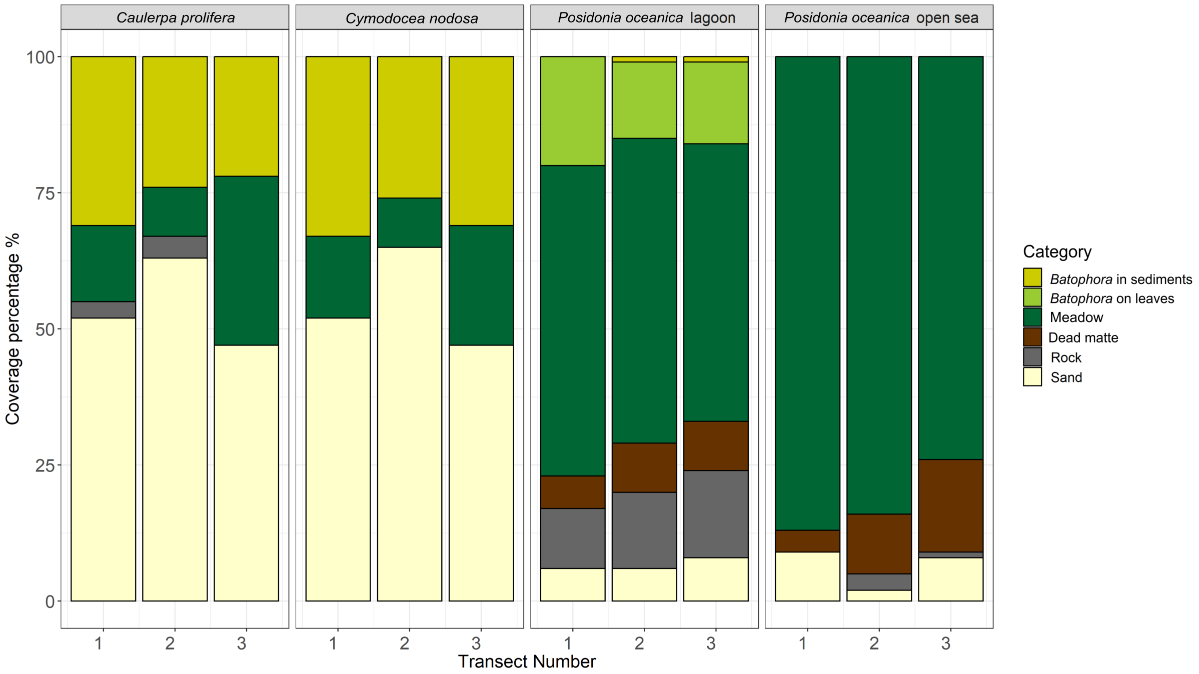

Supplement: S2 Fig — (PDF) [file pone.0338173.s002.pdf]
