# Supplementary material for: Expansion of the exotic macroalga Batophora occidentalis in Posidonia oceanica meadows and other native benthic habitats
Source: PLoS One. 2026 Jul 20;21(7):e0338173. doi: 10.1371/journal.pone.0338173 (PMC13384322; doi:10.1371/journal.pone.0338173)

FIGURE S3. A *Batophora* stalk growing on a leaf of *P. oceanica* from the quadrant samples collected from the meadows outside the lagoon in the open sea. Photo credit: Silvia Paoletti

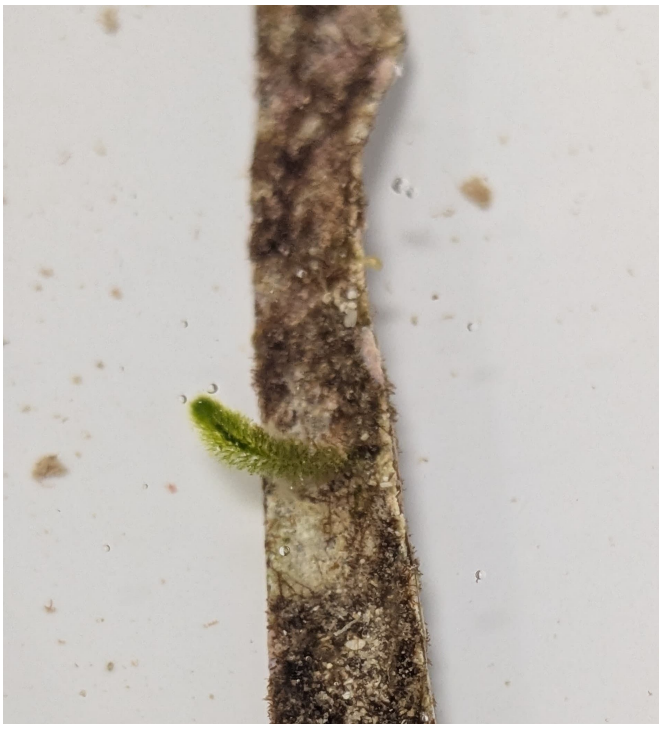

Supplement: S3 Fig — Photo credit: Silvia Paoletti. (PDF) [file pone.0338173.s003.pdf]
