# Supplementary material for: Expansion of the exotic macroalga Batophora occidentalis in Posidonia oceanica meadows and other native benthic habitats
Source: PLoS One. 2026 Jul 20;21(7):e0338173. doi: 10.1371/journal.pone.0338173 (PMC13384322; doi:10.1371/journal.pone.0338173)

FIGURE S4. Four cases where *Batophora* was found attached on leaves of macrophyte species other than *Posidonia oceanica*. **A**) Specimen found anchored to the stolon of *Caulerpa prolifera* (indicated with a red arrow); **B**) specimen found attached to a leaf of *Caulerpa prolifera*; **C**) specimen found attached to a leaf of *Halimeda tuna* within the *C. prolifera* meadows; and **D**) specimen found attached to a leaf of *Cymodocea nodosa*. Photo credit: Silvia Paoletti

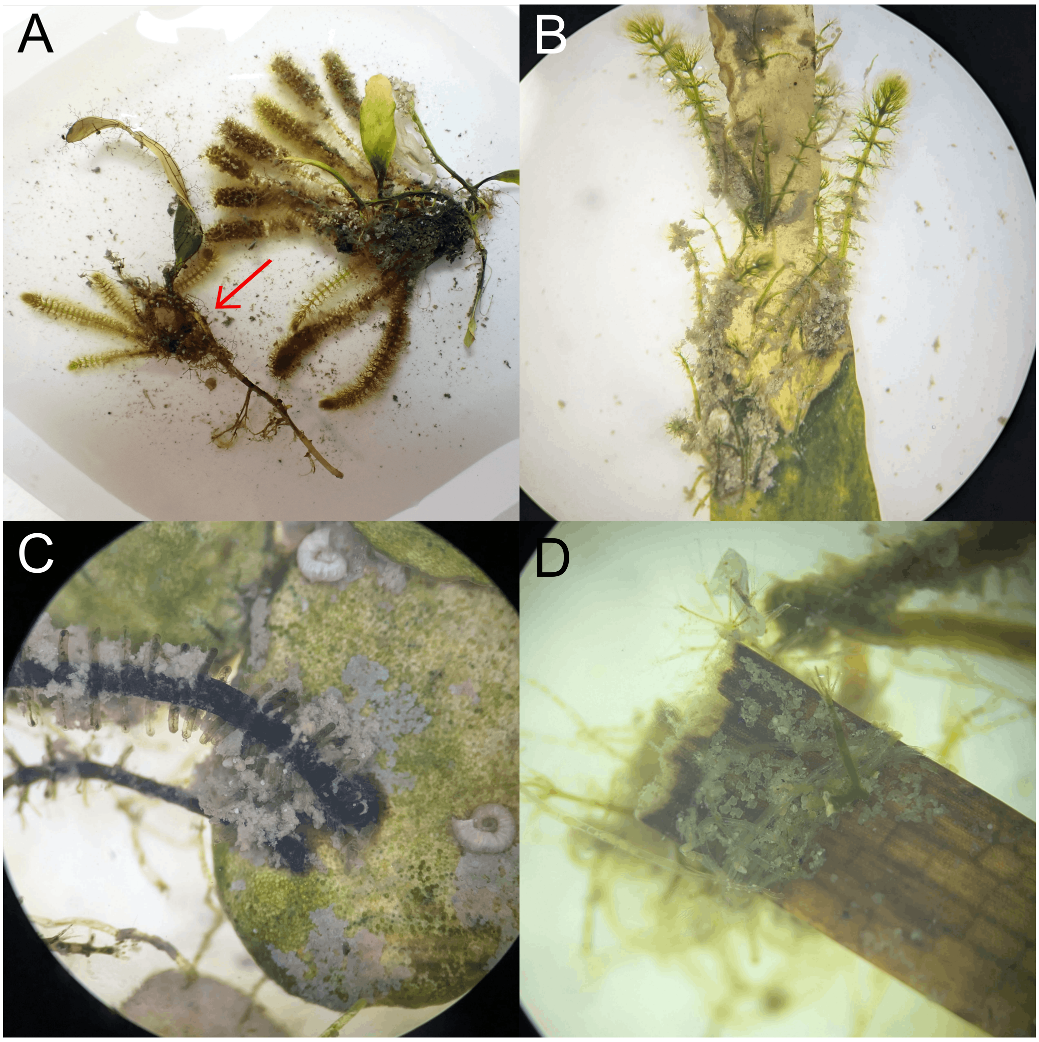

Supplement: S4 Fig — A) Specimen found anchored to the stolon of Caulerpa prolifera (indicated with a red arrow); B) specimen found attached to a leaf of Caulerpa prolifera; C) specimen found attached to a leaf of Halimeda tuna within the C. prolifera meadows; and D) specimen found attached to a leaf of Cymodocea nodosa. Photo credit: Silvia Paoletti. (PDF) [file pone.0338173.s004.pdf]
